# Absence of major epigenetic and transcriptomic changes accompanying an interspecific cross between peach and almond

**DOI:** 10.1093/hr/uhac127

**Published:** 2022-05-26

**Authors:** Carlos de Tomás, Amélie Bardil, Raúl Castanera, Josep M Casacuberta, Carlos M Vicient

**Affiliations:** Centre for Research in Agricultural Genomics CSIC-IRTA-UAB-UB, Campus UAB, Edifici CRAG, Bellaterra, Barcelona 08193, Spain; Institut écologie et environnement (INEE), CNRS, Montpelier, France; Centre for Research in Agricultural Genomics CSIC-IRTA-UAB-UB, Campus UAB, Edifici CRAG, Bellaterra, Barcelona 08193, Spain; Centre for Research in Agricultural Genomics CSIC-IRTA-UAB-UB, Campus UAB, Edifici CRAG, Bellaterra, Barcelona 08193, Spain; Centre for Research in Agricultural Genomics CSIC-IRTA-UAB-UB, Campus UAB, Edifici CRAG, Bellaterra, Barcelona 08193, Spain

## Abstract

Hybridization has been widely used in breeding of cultivated species showing low genetic variability, such as peach (*Prunus persica*). The merging of two different genomes in a hybrid often triggers a so-called “genomic shock” with changes in DNA methylation and in the induction of transposable element expression and mobilization. Here, we analysed the DNA methylation and transcription levels of transposable elements and genes in leaves of *Prunus persica* and *Prunus dulcis* and in an F1 hybrid using high-throughput sequencing technologies. Contrary to the “genomic shock” expectations, we found that the overall levels of DNA methylation in the transposable elements in the hybrid are not significantly altered compared with those of the parental genomes. We also observed that the levels of transcription of the transposable elements in the hybrid are in most cases intermediate as compared with that of the parental species and we have not detected cases of higher transcription in the hybrid. We also found that the proportion of genes whose expression is altered in the hybrid compared with the parental species is low. The expression of genes potentially involved in the regulation of the activity of the transposable elements is not altered. We can conclude that the merging of the two parental genomes in this *Prunus persica* x *Prunus dulcis* hybrid does not result in a “genomic shock” with significant changes in the DNA methylation or in the transcription. The absence of major changes may facilitate using interspecific peach x almond crosses for peach improvement.

## Introduction

Interspecific hybridization is a highly relevant process in plant evolution and breeding, as it can result in phenotypic changes and sexual isolation and be at the origin of new species [1]. Hybridization results in the combination of diverged and novel genes, which can have strong consequences on the phenotype [2]. Hybridization can also induce epigenetic changes, including changes of DNA methylation and in the populations of small RNAs [2]. The genomic changes frequently induced by merging two different genomes can be so wide that they have been frequently referred to as “genomic shock” [3]. For example, important changes in gene expression have been observed in interspecific crosses of species of *Senecio, Tragopogon* or *Gossypium* [4, 5, 6]. On the other hand, structural genome changes have also been reported, in particular the activation of transposable elements (TEs). The transcriptional activation of TEs has been reported in interspecific crosses of, for example, *Spartina*, *Solanum* or *Nicotiana* [7, 8, 9]. Transpositional activation of different TEs was also reported in rice introgression lines derived from crosses with *Zizania latifolia* [10] and increases in TE copy number has been reported in interspecific hybrids of *Helianthus* and *Aegilops* [11, 12]. The activation and mobilization of TEs after hybridization can induce important genome changes through many mechanisms [2], in line with Barbara McClintock ideas of TEs as controller elements helping to reorganize the genome to overcome stress situations [13].

TE activity is tightly controlled by epigenetic mechanisms and DNA methylation is the most obvious and frequent chromatin modification associated to TE silencing [14]. The mutation of different enzymes responsible of DNA methylation and chromatin modification results in a decrease of DNA methylation and induces the activation of plant TEs [15]. Similarly, some biotic and abiotic stresses can result in a decrease of the DNA methylation and can activate the TE transcription and mobilization [16]. The merging of two different genomes in allopolyploids can also induce changes in DNA methylation and gene expression, with important consequences on the phenotype [17]. For example, changes in DNA methylation at the *CONSTANS-LIKE2* gene have an impact on the flowering time in domesticated cotton allotetraploid species [18], and changes in histone modifications in Arabidopsis hybrids and allopolyploids results in an increased biomass, vigour and in starch accumulation [19]. Massive changes in DNA methylation in TEs have been observed in newly formed hybrids, as, for example, in wheat allohexaploid [20]. A decrease in 24-nt small RNAs, which are responsible for DNA methylation, has been shown in F1 allopolyploids between *Arabidopsis thaliana* and *Arabidopsis arenosa* and in intraspecific hybrids of *Arabidopsis thaliana* [21, 22]. Therefore, the merging of two genomes can modify the epigenetic silencing of TEs and frequently results in TE activation that can induce further changes in the genome. However, examples where the merging of two different genomes does not result in changes in TE activity and genome structure have also been reported, for example, in crosses between *Arabidopsis thaliana* and *Arabidopsis lyrata* [23]. TE proliferation has also been reported to be rare in natural *Helianthus* hybrids, despite their widespread transcriptional activity [24]. The reasons for this unpredictable outcome of the merging of two different genomes, and in particular, on TE activation, are not known but it could be related to the level of genome divergence between the two progenitors [2, 7].

Peach (*Prunus persica*) is one of the best-characterized species among the family *Rosaceae* and an important stone fruit crop [25]. Peach does not have a functional gametophytic self-incompatibility system and mainly behaves as self-pollinating, and consequently, it shows low levels of genetic diversity [26]. For this reason, breeders have started to explore the possibility to use other *Prunus* species as an additional source of variability [27]. Almond (*Prunus dulcis*) is one of the closest species to peach, both belonging to the subgenus *Amygdalus* [28]. Peach and almond are diploid (2n = 2x = 16) and have relatively small genomes (about 300 Mbp) which has been sequenced [25, 29]. The two genomes show a high level of similarity and are mainly syntenic [29]. Most almond cultivars are self-incompatible and the almond genome is seven times more variable than peach [30]. Peach and almond can be crossed to produce hybrids that are frequently fertile [31]. In consequence, almond has been considered as an interesting source of novel alleles for peach breeding [27]. Here we investigated to what extend the crosses of peach and almond result in the activation of TEs that could lead to a “genomic shock”.

## Results

### A**nalysis of the potential changes in the DNA methylation of the transposable elements in the peach x almond hybrid**

**Figure 1:**
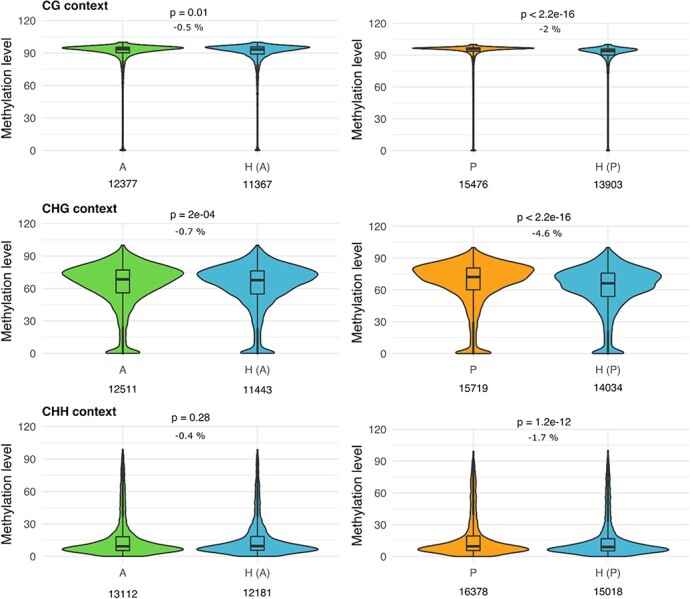
**Distribution of the DNA methylation levels in the transposable elements.** Violin plots for the distribution of mean methylation levels (expressed in percentage) for different methylation sites (CG, CHG and CHH) in transposable elements of the genomes of almond (A, in green), peach (P, in orange) and the interspecific hybrid (H, in blue). H (A) and H (P) represent the methylation levels of the hybrid computed using Almond or Peach genome, respectively. Statistical significance of the differences between parentals and the hybrid is presented (Wilcoxon test p-value), along with the mean methylation differences (average % of methylation in the hybrid minus average % of methylation in the parental). The numbers in the bottom represent the total number of elements analysed in each case.

TE activity is tightly controlled by epigenetic mechanisms [14] and DNA methylation is the most obvious and frequent chromatin modification associated to TE silencing [15]. In consequence, we decided to analyse the TE methylation levels in a peach x almond F1 hybrid and compare them with that of the two parental genomes. Therefore, we performed whole genome bisulphite sequencing of DNA extracted from leaves of almond, peach and an almond x peach F1 hybrid. In both peach and almond, more than half of the annotated TEs were covered by mapped reads in, at least, 25% of their length (59% in almond and 65% in peach) and were included in downstream analyses (Table 1). In order to analyze the methylation of TEs in a peach x almond hybrid we mapped the reads from the hybrid to both parental genomes independently. The sequence of the genomes of peach and almond is highly similar, with an average identity of 97.99% (20 SNPs/Kb) in regions aligning 1:1 [29]. We used mapping parameters allowing the cross-hybridization of most reads from each one of the two subgenomes of the hybrid to each of the parental genomes (see material and methods section). When the reads of the hybrid were mapped onto the almond reference genome, 39% of the TEs were covered by uniquely mapped reads on at least the 25% of their length, whereas this percentage was higher (50%) when they were mapped onto the peach genome. A global analysis of TE methylation in both peach and almond and in their hybrid shows that the patterns of DNA methylation are in general similar (Figure 1). As commonly found in plants [32] the highest mean methylation was observed in CG context in both the parents and in the hybrid (about 90%, in average), followed by the CHG context (about 60%, in average) and the CHH context (about 15%, in average). A comparison of the levels of methylation in peach and almond genomes shows a small although significant demethylation in the hybrid in all contexts in comparison to the two parental except in the comparison to almond in CHH context (Figure 1). The levels of DNA methylation in the hybrid were compared to both peach and almond independently. In all the cases in the parental genomes the TEs showed a higher level of methylation than in the hybrid, but the differences were always small, with a maximum of 4.6% when comparing to peach genome (CHG context) and 0.7% when comparing to almond genome (CHG context) (Figure 1).

**Table 1:** **Percentage of TE elements covered in at least 25% of their annotation in the DNA methylation analyses.** In the hybrid, the TE elements were compared to the almond and peach genomes separately

	**Peach**	**Almond**	**Hybrid (Peach ref.)**	**Hybrid (Almond ref.)**
**Total**	65	59	50	39
**LTR retro.**	68	61	55	42
**LINE**	92	82	83	69
**MITE**	52	53	35	31
**TIR**	66	55	52	37

**Figure 2:**
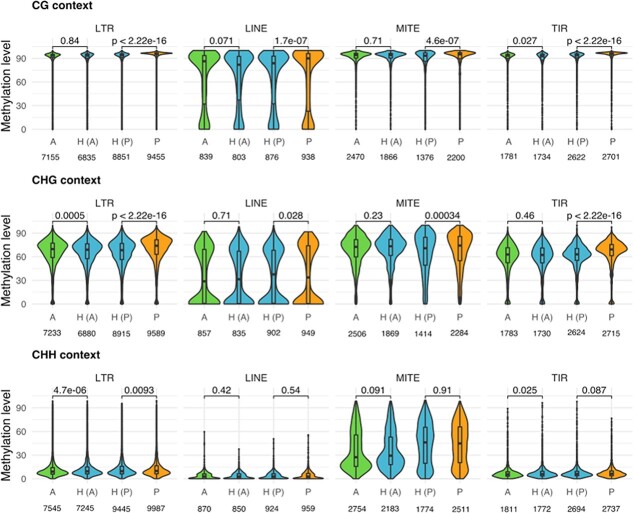
**Distribution of the DNA methylation levels in the transposable elements separated by categories.** Violin plots for the distribution of mean methylation levels (expressed in percentage) for different methylation sites (CG, CHG and CHH) in different classes of transposable elements separately (LTR retrotransposons, LINEs, MITEs and TIRs) in the genomes of almond (A, in green), peach (P, in orange) and the hybrid (H, in blue). H (A) and H (P) represent the methylation levels of the hybrid computed using Almond or Peach genome, respectively. Statistical significance of the differences between parentals and the hybrid is presented (Wilcoxon test p-value). The numbers in the bottom represent the total number of elements analysed in each case.

We then analysed separately the four most abundant classes or TEs present in peach and almond [29] (Figure 2): LTR-retrotransposons, LINEs, MITEs and TIRs. In general, the levels of methylation comparing the hybrid with the parental genomes are very similar with a maximum difference of 5,1% (TIR, Peach vs Hybrid, CHG). The levels of methylation for LTR retrotransposons in the three genomes are similar to the ones obtained for the total TEs (Figure 1). This result was expected due that LTR retrotransposons represent most of the genome space occupied by TEs. The methylation levels of the hybrid are similar to the two parentals except in the case of CHG which is 4,8% higher in peach than in the hybrid. LINE methylation shows a bimodal distribution, with average levels smaller than in the LTR retrotransposons, especially in the CG and CHG contexts. In the case of the MITEs, the average levels of methylation were similar to the LTR retrotransposons in the CG and CHG contexts, but significantly higher in CHH. This is similar to what has been previously found in different rice tissues [33]. When comparing the hybrid with the parental genomes, we observed that MITEs in peach are more methylated than in the hybrid in the CG and CHG contexts, but equally methylated in CHH. No significant differences were found when comparing MITE methylation in almond and the hybrid in any of the three contexts. Finally, the average levels of methylation in TIRs are similar to LTR retrotransposons with only a lower overall methylation in the CHG context of about 10–15%. Comparing the levels in the hybrid with the level in almond, they are very similar. The TIR methylation levels in peach are a slightly higher than in the hybrid (5,1% in the CHG context). In conclusion, we observed differences in the methylation levels of the different classes of TEs but only minor differences when comparing the genomes of the hybrid with the genomes of the two parentals. In spite of these minor differences, the methylation profile of the hybrid was more similar to the almond parental, with only a few exceptions such as LINEs in CHG or MITEs in CHH context.

### Analysis of the differentially methylated regions in the peach x almond interspecific hybrid

The absence of major differences in DNA methylation between parental genomes and the hybrid does not discard the existence of local specific differences that could affect the transcriptional activities of some specific TEs. We therefore decided to analyse the possible presence of differentially methylated regions (DMRs) between the parental lines and the hybrid. We quantified the presence of DMRs in genes, the upstream regions of genes and in LTR retrotransposons comparing the hybrid with the two parental genomes (Table 2). We concentrated on LTR-retrotransposons, as these are the most prevalent TEs in peach and almond genomes [29]. In all the cases, the percentages of DMRs with higher methylation in the hybrid respect to the parents are always around 50%, with a maximum difference of 59% in LTR retrotransposons.

**Table 2:** **DMRs found when comparing the hybrid genome with the parental almond and genomes.** We distinguish between the three different targets of methylation (CG, CHG and CHH), three different regions of the genome (LTR retrotransposons, genes and upstream genes) and those DMRs showing higher methylation in the hybrid (Hybrid > Almond; Hybrid > peach) and showing higher methylation in the parental genome (Almond > Hybrid; Peach > Hybrid). Genes means the region between the ATG and the stop codon (including introns). Upstream genes means the 1000 bp upstream the ATG

**Hybrid vs Almond**									
	**CG**			**CHG**			**CHH**		
	**Total**	**Hybrid > Almond**	**Almond > Hybrid**	**Total**	**Hybrid > Almond**	**Almond > Hybrid**	**Total**	**Hybrid > Almond**	**Almond > Hybrid**
**LTR retro.**	37	41%	59%	209	49%	51%	596	59%	41%
**Genes**	837	54%	46%	527	52%	48%	978	53%	47%
**Upstream genes**	131	46%	54%	154	50%	50%	434	52%	48%
**Hybrid vs Peach**									
	**CG**			**CHG**			**CHH**		
	**Total**	**Hybrid > Peach**	**Peach > Hybrid**	**Total**	**Hybrid > Peach**	**Peach > Hybrid**	**Total**	**Hybrid > Peach**	**Peach > Hybrid**
**LTR retro.**	41	41%	59%	765	45%	55%	722	52%	48%
**Genes**	1319	52%	48%	697	56%	44%	839	53%	47%
**Upstream genes**	254	55%	45%	235	54%	46%	672	54%	46%

**Figure 3:**
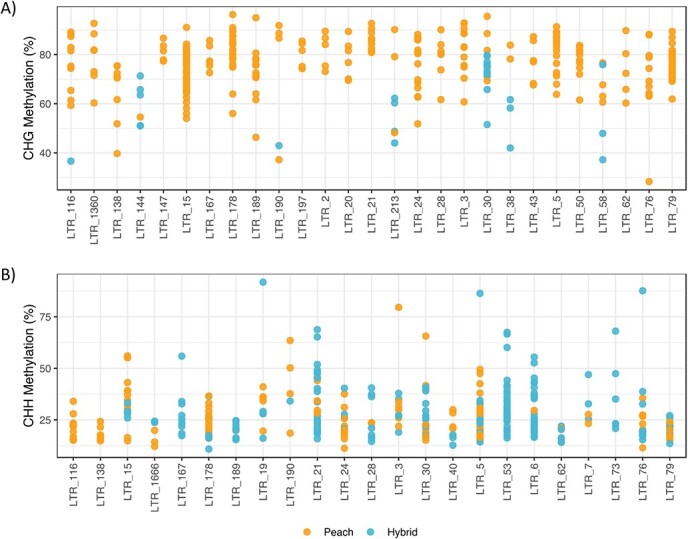
**Differentially methylated regions in LTR retrotransposons when comparing peach and hybrid genomes.** Methylation levels of DMRs in the LTR retrotransposon families comparing peach with the hybrid in CHG (A) and CHH (B) contexts. Blue dots indicate that the DMR is more methylated in the hybrid. Orange dots indicate that the DMR is more methylated in peach. Only families with at least 5 DMRs are shown.

When we concentrate in the LTR retrotransposon families with a higher number of DMRs (Figure 3) we found that in some cases most of the DMRs of the LTR-retrotransposons family correspond to demethylation in the hybrid. This is particularly true in the case of the CHG context when comparing peach with the hybrid (Figure 3A), but also, in minor intensity, in the CHH context (Figure 3B) or when comparing almond with the hybrid (Supplementary Data 1).

### Analysis of the potential changes in the transcription of the transposable elements in the peach x almond hybrid

**Figure 4:**
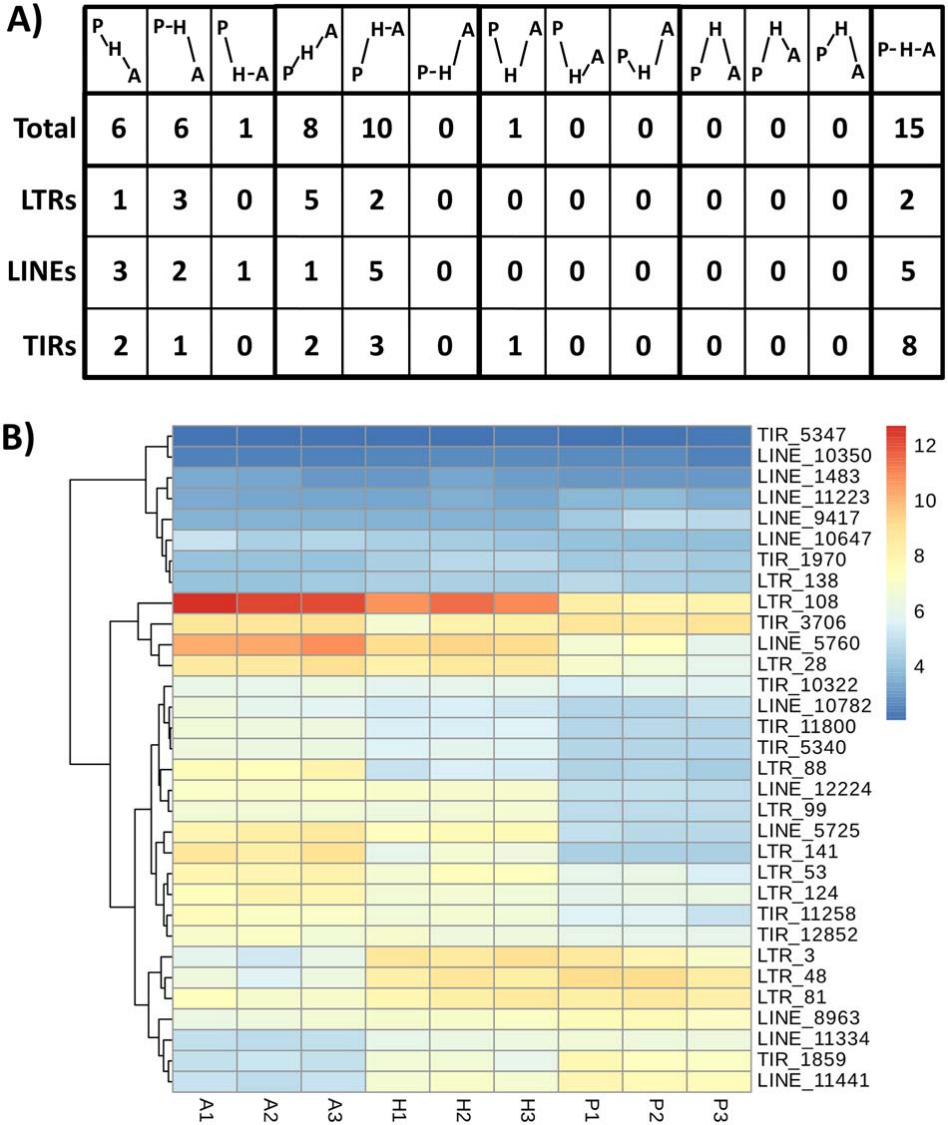
**Transcriptomic analysis of transposable elements in leaves of almond, peach and the hybrid**. A) Patterns of transcription of the TE families. Vertical higher position indicates more transcription (P, peach; H, hybrid; A, almond). Total means the total number of families in peach and almond genomes containing at least one full-length element. B) Heat-map of the transcription levels of the TE families showing expression in at least one of the genotypes. Higher expression is indicated in red and lower expression in dark blue. We show the results of three replicates per sample.

To study the potential activation of TEs by the interspecific cross, we performed an RNA-seq analysis of the expression in mature leaves of almond, peach and the hybrid. We analysed the possible expression of LTR retrotransposons, LINEs and TIRs (Figure 4A). We found significant transcription levels for 47 TE families: 13 LTR retrotransposon, 17 LINE and 17 TIR. Among them, we found significant differential transcription between the almond, peach and/or the hybrid in 32 families (Figure 4A and B): 11 LTR retrotransposon, 12 LINE and 9 TIR. In most of the cases, the differential expression is due to differences between peach and almond and the expression in the hybrid is intermediate. In 18 families the expression was significantly higher in almond than in peach, and in 13 was the opposite. Only one of the TE families showed lower significant expression in the hybrid than in the two parental (TIR_3706) and none was expressed at a higher level than in the two parental species.

A more detailed analysis of the transcribed LTR retrotransposon families showed that in one case (LTR_99) only the almond genome contains full-length copies and this is correlated with a higher expression in almond (Table 3). In 8 of the other 10 transcribed families the species with higher levels of transcription is the one with higher copy number and, in the cases in which that does not happen (LTR_48 and LTR_124), the copy numbers are very similar. On the other hand, all the transcribed LTR retrotransposon families except one (LTR_138) contain relatively young copies with an estimated age of 1,4 Mya or less. In all the families, the parental species with the younger element is the one showing higher transcription (Table 3).

**Table 3:** **Transcribed LTR retrotransposon families.** A, almond; P, peach; H, hybrid

**Family**	**Type**	**Pattern of transcription**	**Number of full copies**		**Minimal estimated time of insertion (Mya)**	
			**Almond**	**Peach**	**Almond**	**Peach**
3	Gypsy	A < (P = H)	11	27	1.7	0.0
28	Gypsy	(A = H) > P	16	10	1.7	0.6
48	Copia	A < (P = H)	26	23	0.0	0.0
53	Copia	A > H > P	26	12	0.0	0.0
81	Copia	A < (P=H)	7	8	1.4	1.4
88	Copia	A > (H = P)	10	5	0.8	0.8
99	Copia	(A = H) > P	3	0	1.4	-
108	Gypsy	A > H > P	14	6	0.0	0.5
124	Unclassified	A > H > P	3	4	10.4	4.3
138	Unclassified	A < H < P	4	11	0.8	0.6
141	Copia	A > (H = P)	5	2	1.3	4.6

**Figure 5:**
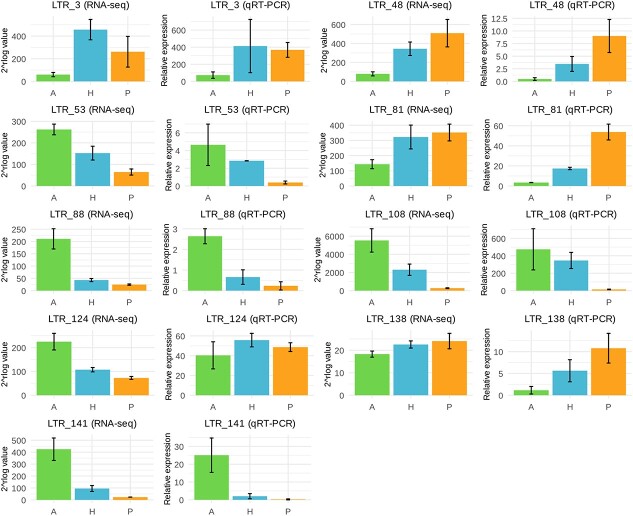
**Transcription of LTR retrotransposon families in leaves of almond, peach and the hybrid.** Transcription levels of LTR retrotransposons in leaves of almond (A, green), peach (P, orange) and the F1 hybrid (H, blue) analysed by qRT-PCR and RNA-seq. The LTR retrotransposon family is indicated in the top.

To validate the RNA-seq data we performed qRT-PCR analysis for nine of the LTR retrotransposon families differentially expressed and the results confirmed their expression profile showing a general agreement with the transcript abundance estimated by RNA-seq (Figure 5).

Next, we tried to identify which of the copies of the differentially transcribed LTR retrotransposon families were the ones that produce transcripts in leaves. For most of the LTR retrotransposon families with differential expression several copies are expressed but from four of them we were able to determine a single copy responsible of all, or most, of the transcription, being in all cases recent insertions present close to genes or inside a gene. In the family LTR_3 the expressed copy is in chromosome 6 of peach (27139890–27 148 350), is estimated to be 1,8 Mya old and is located inside a gene (in an intron) in the same orientation. In the family LTR_81 the expressed copy is in the chromosome 3 of peach (19702391–19 706 564), is estimated to be 1,9 Mya old and is located 3,2 kB from the closest gene. In the family LTR_124 the expressed copy is in the chromosome 7 of almond (21257163–21 260 133), is estimated to be 10,4 Mya old and is located inside a gene (in an intron) in the same orientation. Finally, in the family LTR_141 the expressed copy is in the chromosome 8 of almond (10143038–10 147 556), is estimated to be 1,3 Mya old and is located 35 bases downstream a gene but in opposite orientation.

All these data suggest that the merging of the genomes of peach and almond in a hybrid does not greatly deregulate the expression of TEs and that the differential expression of TEs between these two genomes is mainly due to the presence or absence of transcriptional active copies in each of them.

### Analysis of the potential changes in gene transcription in the peach x almond hybrid

**Figure 6:**
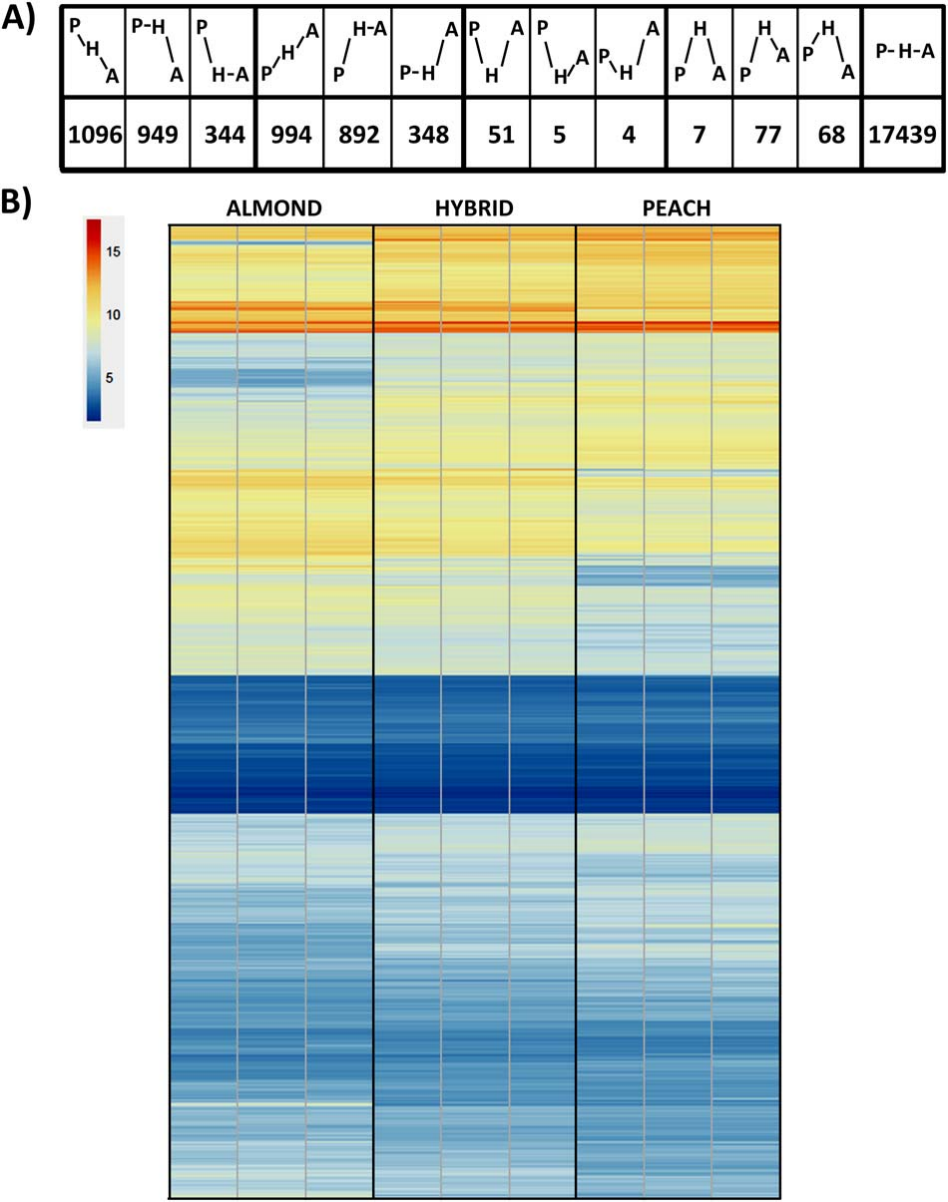
**Transcription of genes in leaves of almond, peach and the hybrid.** Transcriptomic analysis of the genes in leaves of almond, peach and the hybrid. A) Patterns of transcription of the genes. Vertical higher position indicates more transcription (P, peach; H, hybrid; A, almond). B) Average transcription levels of the genes showing significant differential expression in peach, the F1 hybrid and almond. Higher expression is indicated in red and lower expression in dark blue.

We analysed the possible changes in gene expression in the hybrid (Figure 6). As already mentioned, the peach and almond genome show a high degree of sequence identity (mean of 97.99% in regions aligning 1:1, [27]. Therefore, to facilitate the comparison of the level of expression, we decided to map the RNA-seq reads from peach, almond and the hybrid to a single gene model dataset, that of peach or, alternatively, that of almond. A global comparison of the expression levels of 13 620 genes orthologous between peach and almond (see methods for parameters used to define orthology), showed an almost perfect correlation between the two deduced profiles (Pearson correlation coefficient = 0.99) (Supplementary Data S2). We present here the results of the analysis performed using the peach gene models (Figure 6) and the almond gene models (Supplementary Data 3). From the 26 873 genes present in the annotated peach genome [34] we found that 22 274 of them are significantly expressed in at least one of the three genotypes (peach, almond and hybrid). For 17 439 genes (78,3%) the levels of transcription were similar in the three genotypes. For 2389 genes (10,7%) the expression was higher in peach respect to almond and in the F1 hybrid the expression was intermediate or similar to one of the two parents. For 2234 genes (10,0%) the expression was higher in almond respect to peach and in the F1 hybrid was intermediate or similar to one of the two parents. In 152 genes (0,7%) the expression was higher in the hybrid than in the two parental and in 60 genes (0,3%) the expression was lower in the hybrid.

Among the genes whose expression is significantly lower in the hybrid there are two genes, Prupe.1G332600.1 and Prupe.1G334500.1 (Table 4), annotated as potentially encoding an “RNA-dependent RNA polymerase, eukaryotic-type” showing sequence similarity to the Arabidopsis RDR1 (AT1G14790), one of the enzymes involved in the production of sRNAs, in the defence against viruses [35] and in gene regulation and DNA methylation [36]. We next analysed other genes possibly involved in DNA methylation encoding DNA methyltransferases or DNA demethylase (Table 4) but none of them showed differential expression among peach, almond and the hybrid.

**Table 4:** Transcription of genes encoding proteins possibly involved in DNA methylation

	**Almond**		**Hybrid**		**Peach**	
	rlog value	SD	rlog value	SD	rlog value	SD
**RNA-dependent RNA polymerase, eukaryotic-type**						
Prupe.1G332600.1	5,89	0,34	5,09	0,16	5,57	0,42
Prupe.1G334500.1	6,11	0,43	5,45	0,19	6,04	0,28
**DNA methyltransferase**						
Prupe.7G183100.1	7,08	0,30	6,64	0,19	6,67	0,30
Prupe.6G011600.1	2,70	0,03	2,71	0,06	2,96	0,15
Prupe.8G038800.1	9,62	0,33	9,21	0,15	8,97	0,15
Prupe.6G322700.1	10,89	0,10	10,90	0,14	10,91	0,26
**DNA demethylase**						
Prupe.7G118000.1	11,37	0,30	11,36	0,13	11,26	0,09
Prupe.7G005000.1	12,34	0,13	12,41	0,15	12,11	0,27
Prupe.6G119100.1	6,48	0,37	6,63	0,10	7,02	0,09

## Discussion

Hybridization is a very relevant and relatively frequent process in plant evolution [2], that has also been used in plant breeding. For example, crosses of different varieties to produce hybrids presenting superior phenotypes as compared with their parents (hybrid vigour) is widely used in crops [37] and interspecific hybridization with crop wild relatives, or highly variable related species, is frequently used to expand the species variability used for breeding [38]. Peach is a self-fertile and naturally self-pollinating species with very low genetic variability [39]. The use in breeding programs of interspecific crosses with other species of the genus *Prunus* has been historically used to increase peach genetic variability [27]. In the last years a growing interest has emerged in the use of these related species for peach breeding, mainly as a source of pathogen resistances [40]. Almond has become an interesting choice for introgressing new genes into peach, mainly due to the high genetic variability present in almond and also for fruit quality traits [27].

In addition to the combination of diverged alleles and different genes, the merging of two different genomes can also be accompanied by epigenetic and structural changes that can be so widespread that have been defined as a “genomic shock” [3]. Here we analysed the possible genetic and epigenetic changes associated with the crossing of peach and almond to produce an interspecific hybrid. Transposons are the primary target of epigenetic mechanisms, and DNA methylation is the main epigenetic modification associated with TE silencing [15]. Therefore, we have compared the DNA methylation of TEs in peach and almond with that of a F1 interspecific hybrid. Our results show that there are no major differences in the methylation of TEs between the two parental species or between both parents and the hybrid. We found some differentially methylated regions that overlap with LTR retrotransposons, the main order of TEs in peach and almond [29]. In some cases, as for the CHG context, and in particularly when comparing the hybrid with peach, most of the DMRs of the same TE family are demethylated in the hybrid, suggesting a possible weakening of the epigenetic silencing and an increased potential for activation associated with the interspecific cross. However, this demethylated trend has no parallel for the CHH context, where different copies of the same LTR retrotransposon family can show hypermethylated and hypomethylated DMRs in the hybrid. Moreover, the analysis of the transcription of the LTR retrotransposons in leaves in the two parents and in the hybrid did not show the reactivation of any of the LTR retrotransposon families after hybridization. In addition, we neither found transcriptional activation of other types of TEs in leaves of the hybrid. These results suggest that the cross of peach and almond did not result in important changes in the regulation of TEs in general and in the LTR retrotransposons in particular. Among the genes that show a reduced expression in the hybrid there are two genes potentially encoding for an RNA-dependent RNA polymerase showing similarity with the RDR1 protein from Arabidopsis, which is involved in the production of sRNAs, viral defence and DNA methylation [35, 36]. However, a closer look at more genes involved in DNA methylation dynamics [41] did not reveal any difference of expression. Only 1% of the genes showed transgressive expression in the hybrid which reinforces the idea that no major genomic changes are induced by the merging of the peach and almond genomes in the hybrid.

There are many examples were interspecific crosses result in genome demethylation and/or TE activation [11, 12]. In consequence, the lack of signs of a “genomic shock” in the peach x almond hybrid may seem surprising. However, not always interspecific crosses result in genome demethylation and/or TE activation as it has been shown, for example, in crosses between *Arabidopsis thaliana* and *Arabidopsis lyrata* [23]. It has been proposed that when merging two different genomes in a hybrid the intensity of the genome rearrangement and TE mobilization could depend on the TE load imbalance and the phylogenetic distance between the parents [7]. Almond and peach are *Prunus* species of the same subgenera, *Amygdalus*, and have diverged only six million years ago [29]. Considering that the mean generation time for these species is 10 years, this explains the high conservation of their genome sequence which is as low as 20 nucleotide substitutions per Kbp [29]. In addition, the two genomes are also very similar in the proportion and types of TEs they content, sharing the majority of TE families and many individual TE insertions [29]. Therefore, the small phylogenetic distance between peach and almond and their shared TE load could be the reason for the absence of a detectable “genomic shock” associated with their interspecific cross.

In conclusion, our work shows that the merging of peach and almond genomes in an interspecific hybrid is not associated with TE reactivation or general alterations in DNA methylation levels and has not a major impact on gene expression. The absence of alterations in the hybrid may facilitate the use of almond as a source of new genetic variability for breeding the low variable peach species.

## Materials and methods

### Plant material and growth conditions

Leaves of *Prunus dulcis* cv Texas, *Prunus persica* cv Early Gold and one interspecific F1 hybrid MB 1.37 were collected from the Experimental Station of Lleida in Gimenells (Catalonia, Spain) kindly provided by IRTA [27]. They were cultivated in the field. Fully expanded leaves were collected at the end of September from normally watered trees at the same time of the day. Seven leaves per genotype were harvested from three replicates per genotype.

### DNA and RNA isolation

High molecular weight genomic DNA was isolated using a sorbitol pre-wash [42] followed by an adapted CTAB method [43]. Total RNA was extracted using the Maxwell RSC Plant RNA Kit and the Maxwell RSC instrument (Promega Corporation, Madison, WI, USA). Complete DNA removal was obtained using the DNA-free DNA Removal Kit (Invitrogen™, Carlsbad, CA, USA).

### Gene and TE reference datasets

Gene annotations were obtained from Genome Database for *Rosaceae* (GDR; https://www.rosaceae.org/). TE library described in [29] was curated to retain only high-confidence TE annotations (based on the presence of structural features, coding domains or homology to known TEs), resulting in a more stringent annotation (Supplementary Data S4, S5 and S6).

### Analysis of DNA methylation

Library preparation was carried out [29], including two bisulfite conversion rounds to ensure high conversion rate. We calculated bisulfite conversion rates by mapping reads to the non-methylated chloroplast genome. The conversion rates were 97% (almond), 98% (hybrid) and 98% (peach). After processing for removal of adapter and low-quality sequences, we obtained between 41.0 and 44.5 million reads for each of the two independent replicates of the three genotypes. Almond and peach trimmed reads were aligned to the corresponding parental genomes. Reads from the hybrid were aligned independently to peach and to almond genomes.

Cytosine methylation was analysed using Bismark v.0.19.1 [44] and SeqMonk software package (v.1.41; Babraham Institute; http://www.bioinformatics.babraham.ac.uk/projects/seqmonk/). We only included cytosine positions that had been sequenced at least three times. Reads were mapped with Bismark keeping only unique best alignments. A read was considered to align uniquely if one alignment exists that had fewer mismatches to the genome than any other alignment (or if there was no other alignment). We used a slightly relaxed mapping threshold (Bismark parameters: -N 1, —score_min L,0,-0.6) to allow the reads from both hybrid subgenomes to map to either peach or almond reference genomes. We used SeqMonk to quantify DNA methylation levels. SeqMonk filters each call position by the degree of coverage to produce weighted methylation values. We focused only on TE copies covered by sequencing reads in at least 25% of their length (Supplementary Data S7).

To identify differentially methylated regions (DMRs) we used the R package DMRCALLER [45] using a sliding window of 50 bp and a 50 bp sliding interval. *P*-value for each window was calculated using Fisher’s exact test. The p-values were adjusted for multiple testing using Benjamini and Hochberg’s method to control the false discovery rate [46]. Bins with fewer than three cytosines in the specified context or < 0.25 difference in methylation proportion between the two conditions or an average number of reads lower than 8 were discarded. Finally, bins that were at less 300 bp were joined.

### RNA sequencing and analysis

The RNA-seq libraries were obtained from 2–4 μg of total RNA. For each parental and hybrid genotype, three biological replicates were collected. The RNA-seq libraries were produced using the Truseq stranded mRNA protocol and were sequenced on Illumina platform NextSeq 500 (2x150 bp, Paired-end). To analyse TE transcription, we used the methodology described in [47] with minor modifications. The most similar genomic copy to each assembled contig was identified as a family representative (Supplementary Data S8), using a length coverage cut-off of 80% for retrotransposons and 40% for DNA transposons. RNA-seq reads were aligned to peach transcript models concatenated with peach and almond representative TEs using Bowtie2 [48] through the RSEM package in default parameters, keeping only reads aligned in the sense strand. Differential expression analysis was performed using DESeq2 with a Log fold-change cut-off of one. False Discovery Rate (FDR) was used for multiple-testing correction. DESeq2 regularized log (rlog) values were used as normalized expression data for heatmap construction.

### Validation of retrotransposon differential transcription

Quantitative RT-PCR analyses were performed using three independent RNA extractions per genotype. The cDNAs were synthesized using SuperScript® III Reverse Transcriptase (Invitrogen™, Carlsbad, CA, USA). The primers for RT-PCR are listed in Supplementary Data S7. The qRT-PCR were performed in a Roche LightCycler II using Roche’s SYBR green Master Mix (Roche Applied Science) with the initial denaturation step of 5 min at 95°C, followed by 40 RT-PCR cycles (10 s at 95°C, 10 s at 56°C, and 10 s at 72°C). The Translation Elongation Factor (TEF2) and the RNA Polymerase II (RPII) were used as internal controls to normalize the expression of the tested LTR retrotransposons. The relative levels of gene expression were calculated using the 2 − ΔΔCt method. The specificity of the primers and their product length were verified by agarose gel electrophoresis. The primers for qRT-PCR are listed in Supplementary Data S9.

The identification of the expressed TE copies was performed using RT-PCR using different sets of primers (Supplementary Data S7). 1 μg of total RNA was reverse transcribed using SuperScript® III Reverse Transcriptase (Invitrogen, Carlsbad, CA, USA). PCR cycling conditions were 2 min at 95°C, followed by 35 cycles of 95°C for 30 s, 30 s at the annealing Tm and 1 min/kb at 72°C, and a final step of 10 min at 72°C. Negative reverse transcriptase and non-template controls were used. PCR results were observed using a 1% (w/v) agarose gel. PCR fragments were extracted using Macherey-Nagel™ NucleoSpin™ Gel and PCR Clean-up Kit (Fisher Scientific, UK), cloned into the pGEM-Teasy plasmid using pGEM®-T Easy Vector Systems kit (Promega Corporation, Madison, WI, USA), introduced into *E.coli* and amplified. 8 colonies were selected per PCR fragment and their inserts were sequenced. Sequences were compared with the parental genomes and the expressed copies were identified only if the amplified sequences were more than 99,5% identical to the genomic sequence.

## Acknownledgements

We thank Pere Arús, Maria José Aranzana and Iban Eduardo for their help in providing us with the plant material and useful discussions. Raúl Castanera holds a Juan de la Cierva Incorporación Postdoctoral fellowship IJC2020–045949-I funded by MCIN/AEI/10.13039/501100011033 and by the “European Union NextGenerationEU/PRTR”. This work was funded by grants from the Spanish Ministerio de Economía, Industria y Competitividad (AGL2016–78992-R/FEDER), Ministerio de Ciencia e Innovación (PID2019-106374RB-I00/AEI/10.13039/501100011033), by “ERDF A way of making Europe” and by the CERCA Programme of the Generalitat de Catalunya. We also acknowledge financial support from the Spanish Ministerio de Economía y Competitividad through the “Severo Ochoa Programme for Centres of Excellence in R&D” 2016–2019 (SEV-2015-0533) and CEX2019–000902-S funded by MCIN/AEI/10.13039/501100011033.

## Author contributions

CdT performed the transcriptomic analyses. AB performed the DNA methylation analyses. RC contributed to the bioinformatic analyses. JMC and CMV conceived the research and designed the experiments. All authors contributed to the final version of the manuscript.

## Data availability

All data used in this study are publicly available. Raw and processed RNA-seq and bisulfite sequencing data has been deposited to NCBI GEO repository under the accession GSE198152. Genome sequences and annotations are available in the Genome Database for Rosaceae (https://www.rosaceae.org/).

## Conflict of interests

All authors declare no conflict of interests.

## Supplementary data

Supplementary data is available at *Horticulture Research* online.

## Supplementary Material

Web_Material_uhac127
