# Supplementary material for: Absence of major epigenetic and transcriptomic changes accompanying an interspecific cross between peach and almond
Source: Hortic Res. 2022 May 26;9:uhac127. doi: 10.1093/hr/uhac127 (PMC9343919; doi:10.1093/hr/uhac127)
Supplement: Web_Material_uhac127 [file web_material_uhac127.zip › Supplementary Data S4 - Annotation statistics of TEs in almond and peach genomes.docx]

Supplemental Data S1 – Annotation statistics of TEs in almond and peach genomes.

| Class | Peach (copies) | Almond (copies) | Peach  (bp) | Almond (bp) | Peach  (% genome) | Almond  (% genome) |
| --- | --- | --- | --- | --- | --- | --- |
| LTR-retrotransposon | 18.807 | 18.751 | 40.018.216 | 35.804.381 | 17,73 | 16,01 |
| LINE | 1.179 | 1.282 | 1.709.736 | 1.970.960 | 0,76 | 0,88 |
| TIR | 5.313 | 4.972 | 20.668.164 | 15.566.752 | 9,16 | 6,96 |
| MITE | 8.754 | 10.466 | 3.046.983 | 3.462.657 | 1,35 | 1,55 |
| Total | 34.358 | 35.801 | 67.495.012 | 58.236.314 | 29,91 | 26,04 |
