# Supplementary material for: Absence of major epigenetic and transcriptomic changes accompanying an interspecific cross between peach and almond
Source: Hortic Res. 2022 May 26;9:uhac127. doi: 10.1093/hr/uhac127 (PMC9343919; doi:10.1093/hr/uhac127)
Supplement: Web_Material_uhac127 [file web_material_uhac127.zip › Supplementary Data S7 - Percentage of TE elements covered by at least 25% of their length - Copy.docx]

Supplemental Data S3 - Percentage of TE elements covered by at least 25% of their length.

| Class | Almond | Peach |
| --- | --- | --- |
| LINE | 82 | 92 |
| LTR-retrotransposon | 61 | 68 |
| MITE | 53 | 52 |
| TIR | 55 | 66 |
