# Supplementary material for: Absence of major epigenetic and transcriptomic changes accompanying an interspecific cross between peach and almond
Source: Hortic Res. 2022 May 26;9:uhac127. doi: 10.1093/hr/uhac127 (PMC9343919; doi:10.1093/hr/uhac127)
Supplement: Web_Material_uhac127 [file web_material_uhac127.zip › Supplementary Data S9 - DNA primers used in this work.docx]

Supplementary Data S5 - DNA primers used in this work.

| Primer name | Sequence | Used in |
| --- | --- | --- |
| P003_F | AAACAACCATGGTTGGAAGC | RT-PCR |
| P003_R | GAGGTGTATTYTTTGGTGAA | RT-PCR |
| P028_F | ATGCATMARTGTGTACCTCA | RT-PCR |
| P028_R | CTAGGAATGRAGTTCATGGA | RT-PCR |
| P048_F | GGCATTTGCTAGYCTYAGTG | RT-PCR |
| P048_R | ACACCATTTTGYTGTRGTGT | RT-PCR |
| P053_F | CTGATTCCTTGCTCATAGCA | RT-PCR |
| P053_R | CAATGAAGAGTYTTGGGTGT | RT-PCR |
| P081_F | ACTCTGGCCCTGTTGAGCAA | RT-PCR |
| P081_R | TATCTCCACATCCTTTGGCC | RT-PCR |
| P088_F | TAATGGTGTCCAATCTGGCT | RT-PCR |
| P088_R | TTACATGAGAAGGGAATGCC | RT-PCR |
| P108_F | GGTTAGATCTCATGAAGGGA | RT-PCR |
| P108_R | GATGCTAGGCTCTGCGTGTA | RT-PCR |
| P124_F | GGATGAAGCTTGGTGTGATG | RT-PCR |
| P124_R | AGATAAGTTGTCCATAGAAC | RT-PCR |
| P138_F | GTTCCTCTTCAATTGGGTCC | RT-PCR |
| P138_R | GCAGCCAAATCAAGAYCATG | RT-PCR |
| P141_F | CCTTTAGCTACTAACCTGGC | RT-PCR |
| P141_R | GTCCTACTCATGCTGTGAAG | RT-PCR |
| P003_M_F | ACYTGGCAGTGTCCAACTCA | qRT-PCR |
| P003_M_R | TCTATAGCCCACTTCATGAG | qRT-PCR |
| P048_F | GGCATTTGCTAGYCTYAGTG | qRT-PCR |
| P048_M_R | AACAYTTTGGYTTRCCCTTG | qRT-PCR |
| P053_F | CTGATTCCTTGCTCATAGCA | qRT-PCR |
| P053_M_R | TCATCGATGATCACTCTCSG | qRT-PCR |
| P081_M_F | GTGGTTCTACTTGCATATGC | qRT-PCR |
| P081_R | TATCTCCACATCCTTTGGCC | qRT-PCR |
| P088_M_F | TGTGTCTCAATTCAGTTGGC | qRT-PCR |
| P088_R | TTACATGAGAAGGGAATGCC | qRT-PCR |
| P108_F | GGTTAGATCTCATGAAGGGA | qRT-PCR |
| P108_M_R | GTTCCTTCCAATTCTTCCAC | qRT-PCR |
| P124_F | GGATGAAGCTTGGTGTGATG | qRT-PCR |
| P124_M_R | CAAAGTCCACYTTCTCCCAT | qRT-PCR |
| P138_F | GTTCCTCTTCAATTGGGTCC | qRT-PCR |
| P138_M_R | GGTTGAACATCCTTAKTTGG | qRT-PCR |
| P141_M_F | GCATCACACATTTTGTRCTC | qRT-PCR |
| P141_R | GTCCTACTCATGCTGTGAAG | qRT-PCR |
| TEF2_F | GGTGTGACGATGAAGAGTGATG | qRT-PCR |
| TEF2_R | TGAAGGAGAGGGAAGGTGAAAG | qRT-PCR |
| RPII_F | TGAAGCATACACCTATGATGATGAAG | qRT-PCR |
| RPII_R | CTTTGACAGCACCAGTAGATTCC | qRT-PCR |
