# Supplementary figures and images for: Absence of major epigenetic and transcriptomic changes accompanying an interspecific cross between peach and almond
Source: Hortic Res. 2022 May 26;9:uhac127. doi: 10.1093/hr/uhac127 (PMC9343919; doi:10.1093/hr/uhac127)

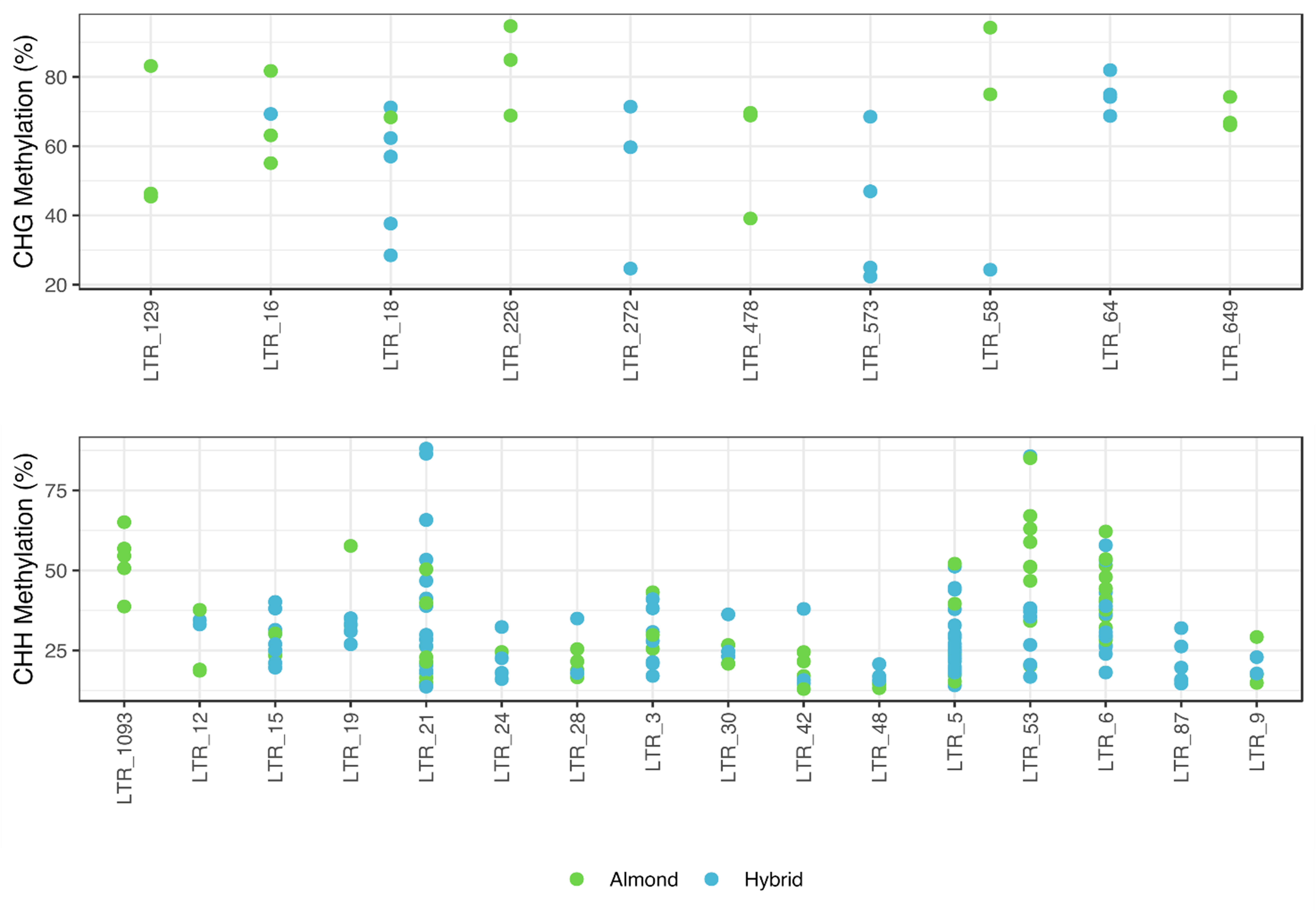

Supplement: Web_Material_uhac127 [file web_material_uhac127.zip › Supplementary Data S1 - DMRs almond LTR retrotransposons.tif]

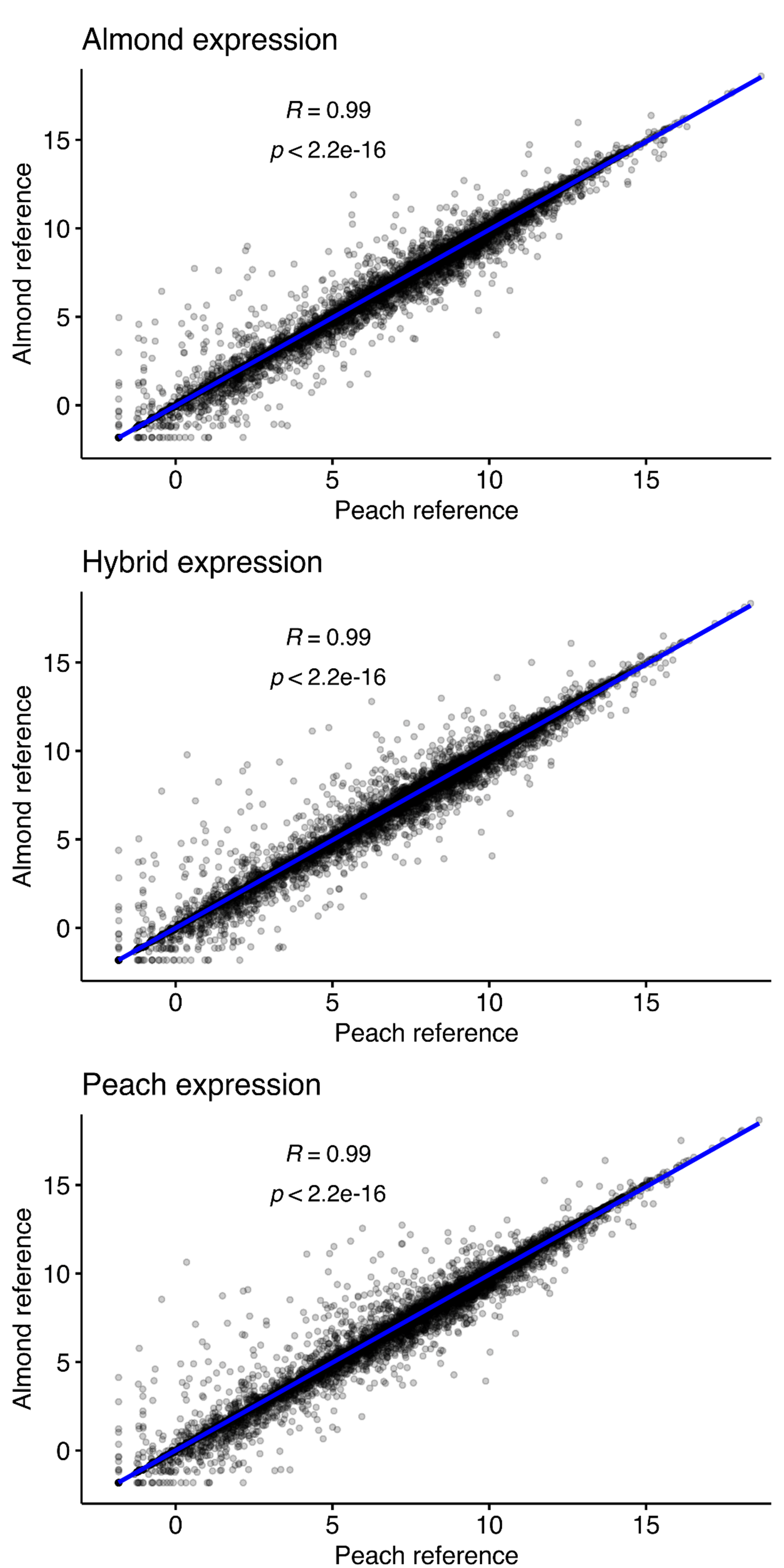

Supplement: Web_Material_uhac127 [file web_material_uhac127.zip › Supplementary Data S2 - Correlation Gene Expression Annotation Peach and Almond.tif]

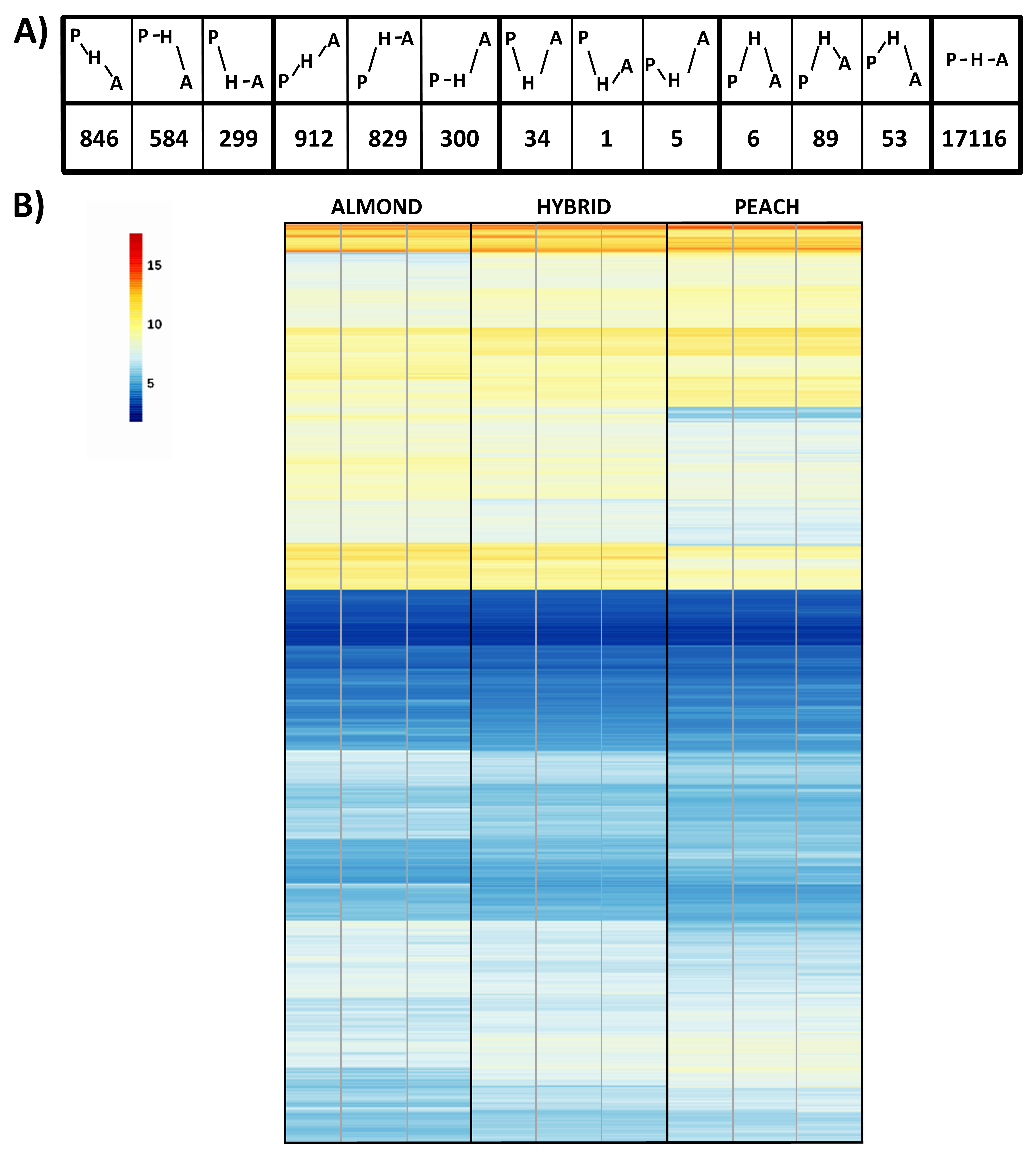

Supplement: Web_Material_uhac127 [file web_material_uhac127.zip › Supplementary Data S3 - Transcription Genes vs Almond annotation.tif]
